# Indocyanine Green as a Single Tracer for Axillary Staging in Breast Cancer: A Retrospective Single-Centre Cohort Study

**DOI:** 10.3390/cancers18101630

**Published:** 2026-05-18

**Authors:** Valentin Ivanov, Usman Khalid, Rosen Dimov, Stefan Ivanov

**Affiliations:** 1 Medical Simulation and Training Center, Medical Faculty, Department of Surgery, University Hospital ‘’Kaspela’’, 4002 Plovdiv, Bulgaria; 2 Medical Faculty, Medical University of Plovdiv, 4002 Plovdiv, Bulgaria; usmankhalid957@gmail.com; 3 Department of Special Surgery, Medical Faculty, Medical University of Plovdiv, 4001 Plovdiv, Bulgaria; rosendimov68@gmail.com (R.D.); sivanov2455@gmail.com (S.I.)

**Keywords:** breast cancer, sentinel lymph node biopsy, indocyanine green fluorescence, axillary staging, fluorescence-guided surgery, lymph node metastasis, single-tracer technique, surgical oncology

## Abstract

**Background/Objectives**: Sentinel lymph node biopsy is central to axillary staging in breast cancer, but conventional mapping often relies on radioisotopes and/or blue dye. Indocyanine green fluorescence has emerged as an alternative, although evidence for its use as a sole tracer in routine practice remains limited. This study evaluated the technical feasibility, lymph node yield, nodal metastasis detection, and short-term clinical outcomes of indocyanine green used as the only tracer for axillary staging in a consecutive single-centre cohort. **Methods**: This retrospective observational cohort study included 260 patients with histologically confirmed breast cancer who underwent axillary surgery at University Hospital Kaspela between 2024 and 2025 under an institutional protocol using indocyanine green as the sole tracer. Indocyanine green-guided mapping was attempted in all patients. For node-focused statistical analyses, a predefined complete-case–cohort of 230 patients was used. Descriptive analyses assessed axillary procedure distribution, lymph node yield, nodal metastasis, and postoperative outcomes. Exploratory multivariable logistic regression was performed to evaluate predictors of nodal metastasis. **Results**: Mapping was successful in 259/260 patients (99.6%). In the complete-case–cohort, sentinel lymph node biopsy was performed in 166/230 patients (72.2%), targeted axillary dissection in 4/230 (1.7%), and axillary lymph node dissection in 60/230 (26.1%). Median overall lymph node yield was 4 (IQR 3–7), but this pooled value reflected heterogeneous axillary procedures and should not be interpreted as sentinel node yield alone. In the clinically node-negative upfront SLNB subgroup, median lymph node yield was 4 (IQR 2.75–5), and nodal metastasis was identified in 22/112 patients (19.6%). Overall, nodal metastasis was identified in 58/230 patients (25.2%), with a median of 2 metastatic nodes (IQR 1–3) among nodal-positive cases. Reoperation for axillary lymph node dissection occurred in 14/230 patients (6.1%). In exploratory multivariable analysis, suspicious biopsied-positive nodes (OR 12.85, 95% CI 3.98–41.52), suspicious non-biopsied nodes (OR 15.58, 95% CI 3.44–70.59), and neoadjuvant therapy (OR 0.31, 95% CI 0.11–0.87) were associated with nodal metastasis; these findings should be interpreted cautiously given the expected clinical relationship between preoperative nodal suspicion and nodal positivity, and the limited number of nodal-positive events. **Conclusions**: Indocyanine green used as a sole tracer demonstrated high technical feasibility within a heterogeneous real-world axillary staging workflow in this single-centre cohort. These findings should be interpreted as implementation-focused feasibility data rather than formal diagnostic validation, given the retrospective design, heterogeneous case mix, and absence of an internal comparator.

## Simple Summary

Sentinel lymph node biopsy is commonly used to assess whether breast cancer has spread to the lymph nodes under the arm. Traditional mapping techniques often require radioactive tracers or blue dye, which can increase logistical complexity and resource requirements. Indocyanine green is a fluorescent dye that may offer a simpler alternative, but evidence on its use as the only tracer in routine clinical practice remains limited. In this study, we evaluated the use of indocyanine green as the sole tracer for axillary staging in a consecutive cohort of breast cancer patients treated at a single centre. We found that this technique achieved a very high mapping success rate and was feasible through routine surgical practice. These findings provide real-world implementation data on indocyanine green-only axillary staging and may help inform future prospective studies evaluating its role in routine clinical practice.

## 1. Introduction

Breast cancer remains a major global health burden and is the most frequently diagnosed cancer worldwide according to recent global cancer statistics [1]. Sentinel lymph node biopsy (SLNB) is central to axillary staging in early breast cancer and is widely incorporated into contemporary practice, with ongoing prospective evidence supporting selective de-escalation of axillary surgery in carefully defined low-risk populations [2,3,4,5].

Standard SLNB mapping has traditionally relied on radiocolloid (e.g., technetium-99m) with or without blue dye. While effective, radiotracer pathways depend on nuclear medicine resources and associated logistics, and blue dye use has been limited in some settings due to adverse reactions and workflow considerations [6,7,8]. These practical constraints have accelerated interest in alternative mapping approaches that can be integrated into routine operating theatre workflows.

Indocyanine green (ICG) fluorescence-guided mapping has emerged as a well-studied technique for SLNB in breast cancer. Contemporary reviews and meta-analyses report high sentinel node identification performance with ICG-based approaches and support its feasibility and safety profile in clinical use [7,9,10,11,12,13,14,15,16]. In addition, prospective, comparative, and meta-analytic evidence has evaluated ICG against technetium-99m, radioisotope, blue dye, methylene blue, or conventional dual-tracer approaches, providing external benchmarks for ICG-based mapping in the studied setting [8,13,14,16,17,18,19,20,21].

There is growing interest in ICG as a single tracer (without radiocolloid or blue dye) as a simplified strategy that may reduce organisational complexity and broaden access where radioisotope pathways are limited. A dedicated single-tracer ICG breast cancer series has reported the feasibility and safety of ICG-only SLNB in clinically node-negative patients [22], while additional feasibility and comparative studies support the broader use of ICG-based SLNB approaches in early breast cancer [18,19,20,21,23,24,25]. Nevertheless, a substantial proportion of the current literature evaluates ICG in combination with radioisotope and/or blue dye, or in comparative mixed-protocol designs, which can limit generalisability to centres adopting ICG-only workflows [6,8].

Therefore, this study aimed to evaluate the technical feasibility, nodal yield, and short-term clinical outcomes of indocyanine green used as a sole tracer for axillary staging within a consecutive single-centre cohort. We acknowledge that ICG-guided SLNB, including ICG-only approaches, has previously been evaluated in dedicated cohorts and comparative studies. Accordingly, the contribution of the present study is incremental: it provides real-world implementation data from a consecutive institutional cohort using an ICG-only axillary mapping workflow, rather than introducing a fundamentally new technique or providing formal diagnostic validation. Rather than assessing diagnostic accuracy metrics such as false-negative rate or long-term oncologic outcomes, this analysis focuses on implementation performance and nodal detection patterns within routine practice.

## 2. Materials and Methods

### 2.1. Study Design and Setting

This retrospective, observational single-centre cohort study was conducted at University Hospital Kaspela, Department of Surgery (Plovdiv, Bulgaria). The study population comprised 260 patients with histologically confirmed breast cancer who underwent axillary surgery at the study centre between 2024 and 2025 and were recorded in a structured institutional database.

All axillary procedures during the study period were performed under an institutional protocol utilising indocyanine green (ICG) as the sole tracer for lymphatic mapping. The final axillary procedure performed (sentinel node biopsy, targeted axillary dissection, or formal axillary dissection) was determined according to standard oncologic indications, including preoperative nodal status and response to neoadjuvant therapy.

For node-focused statistical analyses, a complete-case–cohort of 230 patients was defined based on the availability of predefined variables, including age, imaging tumour size, tumour grade, biological subtype, tumour focality, preoperative axillary status, neoadjuvant therapy, and lymph node count data.

### 2.2. Participants

Adult patients with a diagnosis of breast cancer who underwent axillary surgery at the study centre were included. Consecutive cases documented within the institutional dataset were analysed.

#### Cohort Scope

This consecutive cohort was not restricted to clinically node-negative early breast cancer. Patients with biopsy-proven nodal metastasis and those receiving neoadjuvant therapy were included to reflect the real-world implementation of an ICG-based axillary mapping workflow across the full spectrum of routine surgical practice.

### 2.3. ICG Sentinel Lymph Node Biopsy Technique

Indocyanine green (ICG) fluorescence was used as the sole tracer for lymphatic mapping in accordance with a standardised institutional protocol. ICG was prepared at a concentration of 2.5 mg/mL, corresponding to 25 mg diluted in 10 mL. A fixed total volume of 1.5 mL of ICG solution was administered using a dual peri-areolar intradermal injection technique at two points. Following injection, fluorescence migration towards the axilla was assessed intraoperatively. During the early implementation of the technique, a waiting period of approximately 10 min following injection was commonly used. However, our current practice involves proceeding to sentinel lymph node biopsy once axillary fluorescence is visualised, which typically occurs within 4 min of injection. Prolonged waiting times were avoided to reduce excessive diffusion of ICG within the axilla and fluorescence of multiple lymph nodes. Routine breast massage was not performed. Intraoperative lymphatic mapping and sentinel node identification were performed using an SPY-PHI^®^ fluorescence imaging system (SPY-PHI^®^ fluorescence imaging system (Stryker Corporation, Kalamazoo, MI, USA). Fluorescent lymphatic channels were visualised in real time, and fluorescent sentinel lymph nodes were excised according to standard oncologic surgical principles.

All procedures were performed by the same lead surgeon and institutional breast surgery team using the same fluorescence imaging platform and operative protocol throughout the study period. This study represents the first institutional implementation period for ICG-only axillary mapping at the centre; therefore, the results reflect the real-world adoption of a standardised ICG-only workflow. A formal learning-curve analysis was not performed.

ICG-guided lymphatic mapping was attempted in all patients in the cohort. Mapping success was defined as the intraoperative identification and excision of at least one fluorescent sentinel lymph node, as documented in the operative record.

### 2.4. Data Sources and Variables

Data were extracted from routinely recorded clinical documentation, operative notes, imaging reports, and histopathology records and entered into a structured institutional database. For the purposes of this node-focused analysis, variables were restricted to those relevant to axillary assessment and lymph node outcomes. These included:

• Patient and tumour characteristics: age, number of tumours (focality), imaging tumour size (maximum diameter, mm), tumour grade, biological subtype, preoperative axillary lymph node status, and neoadjuvant therapy.
• Axillary outcomes: final axillary procedure performed (sentinel node biopsy, targeted axillary dissection, or formal axillary dissection), number of lymph nodes removed, and number of metastatic lymph nodes.
• Postoperative outcomes: complications, revision surgery, reoperation for lymph node dissection (ALND), number of nodes removed at ALND, and number of metastatic nodes identified at ALND.

Variables not directly relevant to the node-focused analysis, including breast surgical procedure details and formal tumour staging variables, were not included in the present study.

### 2.5. Outcome Definitions

The primary outcome was the technical feasibility of ICG-guided axillary mapping, defined as the intraoperative identification and excision of at least one fluorescent sentinel lymph node according to operative documentation. Secondary descriptive outcomes included lymph node yield, defined as the total number of lymph nodes removed according to the final axillary procedure performed, and nodal metastasis detection, defined as the presence of ≥1 metastatic lymph node on final histopathology.

This study was not designed to evaluate formal diagnostic accuracy metrics such as sensitivity, specificity, or false-negative rate, as no internal reference standard or dual-tracer comparator was available. Postoperative complications, revision surgery, and reoperation for lymph node dissection (ALND) were recorded from routine clinical documentation within 30 days of the index surgical procedure. Revision surgery reflects any subsequent operative intervention within this period. Reoperation for ALND refers specifically to secondary axillary surgery performed within 30 days following the index operation.

### 2.6. Biological Subtype Classification

Biological subtype was recorded in the institutional dataset using routine clinical classification. For analytical purposes, subtypes were recoded into four clinically relevant categories: Luminal A, Luminal B, HER2-enriched, and Triple-negative. Cases with non-classifiable subtype data were excluded during the definition of the complete-case node-focused analysis cohort.

### 2.7. Ethical Considerations and Reporting Standards

This study utilised retrospectively collected, routinely recorded clinical data and was conducted in accordance with institutional and regulatory requirements. Ethical approval and/or waiver of informed consent were obtained in accordance with institutional requirements. The study design and reporting adhere to the Strengthening the Reporting of Observational Studies in Epidemiology (STROBE) guidelines for observational cohort studies. As a retrospective analysis of routinely recorded clinical data, this study is subject to potential information and documentation bias. Sentinel mapping success was determined from operative records, and subtle technical challenges may not have been captured in standardised form.

For statistical analyses, a predefined complete-case–cohort was used, restricted to patients with available data for all variables included in the node-focused analysis. No imputation was performed. Consecutive case inclusion and use of a standardised institutional operative protocol were intended to reduce selection and procedural heterogeneity.

### 2.8. Statistical Analysis

Statistical analyses were performed using Python (v3.13.11) with the *statsmodels* package. Continuous variables were assessed for normality and are reported as median with interquartile range (IQR), as distributions were non-normal on inspection. Categorical variables are presented as frequencies and percentages. The study population comprised 260 patients who underwent axillary surgery at the study centre. For node-focused statistical analyses, a predefined complete-case–cohort of 230 patients was used, with available data for age, imaging tumour size, tumour grade, biological subtype, tumour focality, preoperative axillary status, neoadjuvant therapy, and lymph node count data. No imputation was performed.

Descriptive analyses of node-focused baseline characteristics, axillary procedure distribution, lymph node yield, nodal metastasis, and postoperative axillary outcomes were performed in the 230-patient complete-case–cohort. Lymph node yield was defined as the total number of lymph nodes removed according to the final axillary procedure performed. Nodal metastasis was defined as the presence of at least one metastatic lymph node on final histopathology.

To address clinical heterogeneity within the cohort, descriptive subgroup analyses were performed according to preoperative axillary status, neoadjuvant therapy, and final axillary procedure. Subgroup outcomes included procedure composition, lymph node yield, nodal positivity, postoperative complications, revision surgery, and reoperation for ALND.

Exploratory logistic regression was performed to evaluate predictors of nodal metastasis. Univariable logistic regression was first performed for each prespecified covariate. A multivariable model was then fitted including age, imaging tumour size, tumour grade, biological subtype, tumour focality, preoperative axillary status, and neoadjuvant therapy. Biological subtype was entered as a categorical variable using Luminal A as the reference category. Tumour grade was dichotomised as G3 versus G2 due to sparse G1 representation. Results are reported as odds ratios (ORs) with 95% confidence intervals (CIs). Given the limited number of reoperations for lymph node dissection events, no multivariable model was fitted for that endpoint to avoid overfitting. All regression analyses were considered exploratory and hypothesis-generating.

## 3. Results

### 3.1. Cohort Characteristics

The node-focused statistical analysis cohort comprised 230 patients with complete data for predefined variables. Median age was 57.5 years (IQR 46.0–68.0). The maximum tumour diameter on preoperative imaging was 18.35 mm (IQR 12.0–23.75). Tumours were predominantly unifocal (196/230, 85.2%), while 34/230 (14.8%) were multifocal or multicentric. Tumour grade was G2 in 192/230 (83.5%) and G3 in 38/230 (16.5%).

Biological subtype distribution included Luminal B in 99/230 patients (43.0%), Luminal A in 95/230 (41.3%), triple-negative disease in 23/230 (10.0%), and HER2-enriched disease in 13/230 (5.7%). On preoperative axillary assessment, 173/230 patients (75.2%) had no suspicious lymph nodes, 24/230 (10.4%) had suspicious biopsied-positive nodes, 21/230 (9.1%) had suspicious biopsied-negative nodes, and 12/230 (5.2%) had suspicious non-biopsied nodes. Neoadjuvant therapy was administered in 70/230 patients (30.4%), while 160/230 (69.6%) did not receive neoadjuvant treatment. Table 1 demonstrates the baseline node-focused clinicopathological characteristics of the predefined complete-case–cohort.

**Table 1:** Baseline node-focused clinicopathological characteristics of the predefined complete-case–cohort (n = 230).

Characteristic	Value
**Age (years), median (IQR)**	57.5 (46.0–68.0)
**Imaging tumour size, max diameter (mm), median (IQR)**	18.35 (12.0–23.75)
**Tumour focality**	
• Unifocal	196 (85.2%)
• Multifocal/multicentric	34 (14.8%)
**Tumour grade**	
• G2	192 (83.5%)
• G3	38 (16.5%)
**Biological subtype**	
• Luminal A	95 (41.3%)
• Luminal B	99 (43.0%)
• HER2-enriched	13 (5.7%)
• Triple-negative	23 (10.0%)
**Preoperative axillary lymph node status**	
• No suspicious nodes	173 (75.2%)
• Suspicious, biopsied-positive	24 (10.4%)
• Suspicious, biopsied-negative	21 (9.1%)
• Suspicious, not biopsied	12 (5.2%)
**Neoadjuvant therapy**	
• Yes	70 (30.4%)
• No	160 (69.6%)

#### Participant Flow

**Figure 1:**
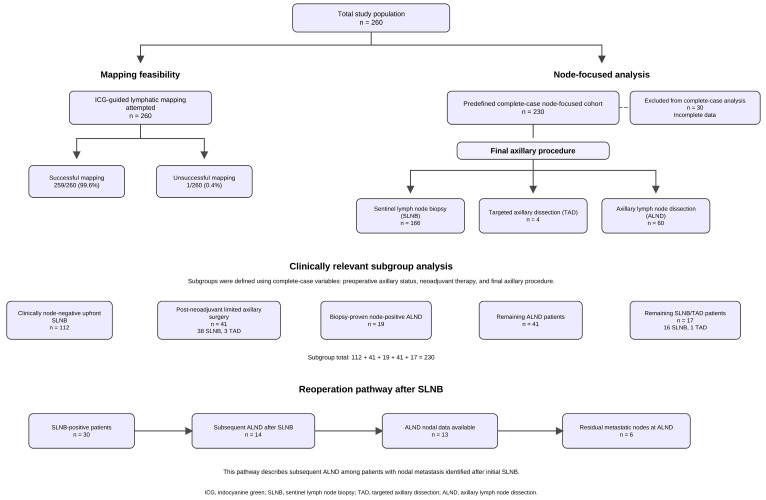
Participant flow, mapping success, and subgroup composition of the node-focused analysis cohort.

A total of 260 patients undergoing axillary surgery between 2024 and 2025 were identified in the institutional dataset and comprised the study population. For node-focused statistical analyses, a predefined complete-case–cohort of 230 patients was used. ICG-guided lymphatic mapping was attempted in all patients in the cohort. Successful mapping was documented in all but one case according to operative records. Within the cohort, sentinel node biopsy was performed predominantly in clinically node-negative cases, targeted axillary dissection was performed in selected patients with documented nodal disease and response to neoadjuvant therapy, and formal axillary dissection was performed in node-positive cases or for other standard oncologic indications. Figure 1 displays the participant flow, mapping success, and subgroup composition of the node-focused analysis cohort.

### 3.2. Axillary Procedure and Lymph Node Yield

The recorded axillary procedure reflected the final surgical management decision based on preoperative and intraoperative assessment. Within the node-focused analysis cohort (n = 230), sentinel node biopsy was performed in 166/230 patients (72.2%), axillary lymph node dissection (ALND) in 60/230 (26.1%), and targeted axillary dissection in 4/230 (1.7%) (Table 2).

ALND cases included patients with preoperative biopsy-proven nodal metastasis, persistent nodal disease following neoadjuvant therapy, or other standard oncologic indications. Targeted axillary dissection was performed in selected patients with documented nodal disease who demonstrated complete clinical response following neoadjuvant therapy and had a previously marked positive lymph node.

Across the cohort, the number of lymph nodes removed, reflecting total nodes excised according to the final axillary procedure performed, had a median of 4 (IQR 3–7). Lymph node yield varied by procedure:

• Sentinel node biopsy: median 4 (IQR 3–5);
• Targeted axillary dissection: median 6 (IQR 4.75–7);
• Axillary lymph node dissection: median 11 (IQR 7.75–18).

ICG-guided lymphatic mapping was attempted in all patients in the study population and was successful in all but one case according to operative documentation. Node yield reflects the total number of lymph nodes removed according to the final axillary procedure performed, rather than sentinel nodes alone.

### 3.3. Nodal Metastasis

Nodal metastasis was identified in 58/230 patients (25.2%). Among nodal-positive cases, the median number of metastatic lymph nodes was 2 (IQR 1–3). Overall axillary outcome summaries are presented in Table 2.

### 3.4. Subgroup Analysis by Axillary Treatment Pathway

To improve the interpretability of outcomes across clinically distinct axillary pathways, a subgroup analysis was performed within the predefined complete-case–cohort according to preoperative axillary status, neoadjuvant therapy, and final axillary procedure. The clinically node-negative upfront SLNB subgroup comprised 112 patients with no suspicious preoperative axillary nodes, no neoadjuvant therapy, and SLNB as the final axillary procedure. In this subgroup, median lymph node yield was 4 nodes (IQR 2.75–5), nodal metastasis was identified in 22/112 patients (19.6%), postoperative complications occurred in 16/112 patients (14.3%), and reoperation for ALND occurred in 11/112 patients (9.8%).

Among post-neoadjuvant patients managed with limited axillary surgery, including SLNB or targeted axillary dissection, 41 patients were identified. Median lymph node yield was 4 nodes (IQR 3–5), nodal metastasis was identified in 7/41 patients (17.1%), complications occurred in 4/41 patients (9.8%), and reoperation for ALND occurred in 3/41 patients (7.3%). In patients with biopsy-proven nodal disease undergoing ALND, median lymph node yield was 11 nodes (IQR 8.5–17.5), nodal metastasis was identified in 11/19 patients (57.9%), complications occurred in 4/19 patients (21.1%), and no patients underwent reoperation for ALND.

The remaining ALND patients accounted for 41 patients and were associated with a median lymph node yield of 11 nodes (IQR 6–18), nodal positivity in 14/41 patients (34.1%), and complications in 10/41 patients (24.4%). Remaining SLNB/TAD patients accounted for 17 patients, with a median lymph node yield of 4 nodes (IQR 2–5), nodal positivity in 4/17 patients (23.5%), and complications in 2/17 patients (11.8%). These subgroup findings confirm that pooled lymph node yield was strongly influenced by inclusion of ALND patients and should not be interpreted as sentinel node yield. Mapping success was reported at the full study-population level because ICG-guided mapping was attempted in all 260 patients and only one mapping failure was documented. Given the single failure event and the use of the complete-case–cohort for subgroup analyses, subgroup-specific mapping success rates were not considered clinically informative. The clinically node-negative upfront SLNB subgroup provides the most relevant comparison with published ICG-only SLNB cohorts, whereas the overall cohort reflects implementation of an ICG-only axillary workflow across heterogeneous real-world surgical indications. Table 3 highlights clinically relevant subgroup analysis according to axillary treatment pathway within the predefined complete-case–cohort.

**Table 3:** Clinically relevant subgroup analysis according to axillary treatment pathway within the predefined complete-case–cohort.

Subgroup	n	Procedure Composition	Lymph Node Yield, Median (IQR)	Nodal Positivity	Complications	Revision Surgery	Reoperation for ALND
Clinically node-negative upfront SLNB	112	112 SLNB	4 (2.75–5)	22/112 (19.6%)	16/112 (14.3%)	15/112 (13.4%)	11/112 (9.8%)
Post-neoadjuvant limited axillary surgery	41	38 SLNB, 3 TAD	4 (3–5)	7/41 (17.1%)	4/41 (9.8%)	3/41 (7.3%)	3/41 (7.3%)
Biopsy-proven node-positive ALND	19	19 ALND	11 (8.5–17.5)	11/19 (57.9%)	4/19 (21.1%)	4/19 (21.1%)	0/19 (0.0%)
Remaining ALND patients	41	41 ALND	11 (6–18)	14/41 (34.1%)	10/41 (24.4%)	9/41 (22.0%)	0/41 (0.0%)
Remaining SLNB/TAD	17	16 SLNB, 1 TAD	4 (2–5)	4/17 (23.5%)	2/17 (11.8%)	2/17 (11.8%)	0/17 (0.0%)

SLNB, sentinel lymph node biopsy; TAD, targeted axillary dissection; ALND, axillary lymph node dissection; IQR, interquartile range. Subgroups were defined using available complete-case variables, including preoperative axillary status, neoadjuvant therapy, and final axillary procedure. The “remaining ALND patients” and “remaining SLNB/TAD patients” categories included patients who did not meet the predefined criteria for the clinically node-negative upfront SLNB, post-neoadjuvant limited axillary surgery, or biopsy-proven node-positive ALND subgroups.

### 3.5. Reoperation for Lymph Node Dissection and ALND Nodal Burden

Reoperation for axillary lymph node dissection (ALND) occurred in 14/230 patients (6.1%) (Table 4). All reoperations followed an initial sentinel node biopsy, corresponding to 14/166 SLNB cases (8.4%). Among patients with nodal metastasis identified on sentinel node biopsy (30 cases), 14 (46.7%) subsequently underwent ALND. ALND-specific nodal data were available for 13 of these patients. Among them, residual metastatic lymph nodes were identified in 6/13 cases (46.2%). The number of metastatic nodes identified at ALND is summarised in Table 4. Only one patient underwent immediate intraoperative conversion from SLNB to ALND due to unsuccessful sentinel node mapping; all other ALND procedures following SLNB were performed based on oncologic indications.

### 3.6. Resection Margins, Complications, and Revision Surgery

Postoperative complications were recorded in 36/230 patients (15.7%). The most frequently observed complication was seroma (n = 22), followed by bleeding (n = 8) and inflammatory wound complications (n = 6). Seroma cases were managed with ultrasound-guided aspiration where appropriate; 12 patients subsequently required revision surgery with drain placement. Inflammatory wound complications were managed with antibiotic therapy in all but one case, which required revision surgery.

Revision surgery was performed in 33/230 patients (14.3%). This included 14 patients who underwent reoperation for ALND after initial SLNB and 19 patients who underwent non-ALND revision surgery. Non-ALND revision surgery included bleeding/haematoma revision in 6 patients, inflammatory wound revision in 1 patient, and seroma revision with drain placement in 12 patients. Therefore, the overall revision surgery rate should be interpreted as a composite endpoint including secondary axillary surgery, seroma-related revision, bleeding-related revision, and wound revision, rather than as a direct measure of wound morbidity alone. All patients had negative final resection margins (R0).

### 3.7. Exploratory Logistic Regression for Nodal Metastasis

Exploratory logistic regression was performed to evaluate associations with nodal metastasis, defined as the presence of at least one metastatic lymph node on final histopathology. Analyses were performed in the predefined complete-case node-focused cohort (n = 230), with 58 nodal-positive events. The results should be interpreted cautiously because the number of events was modest relative to the number of covariates and because the association between suspicious preoperative axillary findings and final nodal positivity is clinically expected. In multivariable analysis, suspicious biopsied-positive nodes (OR 12.85, 95% CI 3.98–41.52, *p* < 0.001), suspicious non-biopsied nodes (OR 15.58, 95% CI 3.44–70.59, *p* < 0.001), and neoadjuvant therapy (OR 0.31, 95% CI 0.11–0.87, *p* = 0.026) were associated with nodal metastasis. Tumour grade (G3 vs. G2) showed a borderline association with nodal metastasis (OR 2.83, 95% CI 0.98–8.17, *p* = 0.055).

Age, imaging tumour size, biological subtype, and tumour focality were not statistically associated with nodal metastasis in this exploratory model (all *p* > 0.05). Results of the regression analyses are summarised in Table 5.

## 4. Discussion

In this consecutive single-centre cohort of 260 breast cancer patients undergoing axillary staging with indocyanine green (ICG) as the sole tracer, fluorescence-guided sentinel lymph node biopsy (SLNB) using the SPY-PHI^®^ system was feasible in routine practice, with successful sentinel mapping documented in 259/260 cases (99.6%). Within the node-focused complete-case–cohort (n = 230), subgroup analysis showed that lymph node yield varied substantially according to the final axillary procedure, with higher yields in ALND-containing groups. Therefore, the pooled median lymph node yield should be interpreted as a workflow-level outcome rather than as a sentinel node yield. The clinically node-negative upfront SLNB subgroup provides the most clinically relevant comparison with published ICG-only SLNB cohorts. As a retrospective analysis of routinely collected institutional data, this study reflects real-world clinical practice but is inherently subject to limitations of retrospective data collection and documentation standardisation.

Contemporary evidence, including dedicated ICG-only cohorts, prospective comparative trials, feasibility studies, reviews, and meta-analyses, has already demonstrated the feasibility and high identification performance of ICG-guided SLNB in breast cancer [6,7,8,9,10,11,12,13,14,15,16,17,18,19,20,21,22,23,24,25]. Therefore, the present study should not be interpreted as introducing a new mapping concept or as providing formal diagnostic validation. Instead, its contribution is incremental and implementation-focused, reporting outcomes from a consecutive single-centre cohort managed under an ICG-only institutional workflow. Because this cohort included patients managed with SLNB, targeted axillary dissection, and ALND based on oncologic indications, including biopsy-proven node-positive disease and post-neoadjuvant settings, the findings should be interpreted as real-world workflow data across heterogeneous axillary indications rather than as a controlled comparison with dual-tracer techniques.

Postoperative outcomes in our cohort were acceptable, with complications predominantly minor (seroma, bleeding, and inflammatory wound complications). Reoperation for ALND occurred in 14/230 patients (6.1%), all following initial SLNB (14/166, 8.4%). Among patients with nodal metastasis identified on SLNB (30 cases), 14 (46.7%) subsequently underwent ALND, with residual metastatic nodes identified in 6/13 cases (46.2%) where nodal data were available. These findings provide additional context for axillary disease burden and surgical decision-making in this cohort.

Pellini et al. reported sentinel node detection and removal in 98.3% of ICG-only cases, while prospective comparative studies such as GREENORBLUE and FLUORO provide external benchmarks for ICG-based approaches in relation to conventional tracer strategies [6,8,22]. These studies are useful for contextualising our findings, but they do not provide an internal comparator for the present cohort. In our study, near-complete mapping success was observed (259/260, 99.6%); however, comparative claims regarding equivalence or non-inferiority to dual-tracer or radioisotope-guided approaches cannot be made because no internal comparator, false-negative rate analysis, or long-term oncologic follow-up was available.

Taken together, the existing literature supports ICG as a feasible and effective fluorescence tracer for SLNB, but the present study should be interpreted as real-world implementation evidence rather than comparative diagnostic validation. ICG-only approaches may reduce reliance on nuclear medicine infrastructure, but direct comparison with dual-tracer strategies was not possible in this retrospective single-centre cohort.

The multivariable regression analysis was retained as an exploratory, hypothesis-generating analysis rather than as a clinical prediction model. The observed association between suspicious preoperative axillary findings and final nodal positivity is clinically expected and should not be interpreted as a novel predictive finding. In addition, the model included multiple covariates relative to 58 nodal-positive events, raising potential concerns regarding precision and stability in line with established considerations for clinical prediction modelling [26]. Accordingly, the regression results are presented only to describe associations within this cohort, and the absence of statistically significant associations for other clinicopathological variables should not be interpreted as evidence of no effect.

This study has several strengths. It evaluates an ICG-only workflow in a large consecutive cohort of 260 patients managed under a standardised institutional protocol, reducing selection bias and procedural heterogeneity. Mapping feasibility was high, and the dataset includes both clinicopathological variables and postoperative outcomes, enabling transparent reporting of nodal yield, nodal metastasis, and reoperation patterns. The use of a predefined complete-case–cohort and cautious interpretation of regression analyses further strengthen methodological transparency.

Several limitations should be acknowledged. First, the retrospective single-centre design introduces potential for unmeasured confounding and limits generalisability. Second, the heterogeneous case mix, while reflective of real-world practice, limits direct comparison with strictly defined validation cohorts. Third, the dataset does not permit formal evaluation of diagnostic accuracy endpoints such as false-negative rate or long-term oncologic outcomes. Fourth, the absence of an internal dual-tracer comparator limits direct comparative inference. Finally, mapping success was determined retrospectively from operative documentation and may not capture subtle technical challenges that would be better characterised in prospective studies with predefined criteria. In addition, although all cases were performed by the same lead surgeon and institutional breast surgery team using a standardised protocol, this cohort represented the first institutional implementation period for ICG-only axillary mapping and was not designed to formally quantify learning-curve effects.

## 5. Future Directions and Implementation

Future research should evaluate ICG-only axillary mapping in prospective multicentre designs with predefined subgroup analyses and clinically relevant endpoints. In particular, clinically node-negative upfront SLNB patients should be analysed separately from post-neoadjuvant and ALND populations, as these groups have different indications, expected lymph node yields, and clinical comparators. Future studies should also include direct comparator arms using radioisotope and/or blue dye where feasible, allowing formal assessment of diagnostic performance, mapping failure, false-negative rate, and oncologic outcomes [6,8,13,14,16,17,18,19,20,21,25,27]. Future prospective studies should standardise and report technical protocol variables, including ICG concentration, injection volume, injection site and plane, injection-to-incision or injection-to-detection interval, massage protocol, fluorescence platform, operator experience, and learning-curve effects.

Beyond technical detection, future work should assess whether ICG-only workflows improve operating theatre logistics, reduce dependence on nuclear medicine infrastructure, and maintain acceptable patient outcomes in routine practice. Implementation endpoints, complication rates, arm morbidity, quality of life, reoperation patterns, and cost or resource-use outcomes should be incorporated alongside mapping success and nodal yield. Particular attention should also be given to the post-neoadjuvant setting, where lymphatic mapping may be more challenging and where protocol optimisation may be needed [28,29,30].

From an implementation perspective, ICG-only mapping may simplify workflow by reducing reliance on preoperative lymphoscintigraphy, nuclear medicine coordination, and radiotracer-based scheduling, which are recognised practical limitations of conventional technetium-based SLNB pathways [31]. However, these potential advantages must be balanced against the requirement for access to a near-infrared fluorescence imaging platform such as SPY-PHI^®^, equipment acquisition or depreciation costs, staff familiarisation, and operating theatre integration. Prior organisational and economic analyses have shown that ICG pathways may streamline surgical workflow, reduce waiting times, and improve scheduling flexibility while also requiring investment in fluorescence-detection equipment [31]. Therefore, the feasibility of ICG-only adoption is likely to depend on local infrastructure, procedure volume, equipment availability, and institutional cost structures.

## 6. Conclusions

In this consecutive single-centre cohort of 260 patients with breast cancer undergoing axillary surgery, indocyanine green used as a sole tracer demonstrated high technical feasibility within a heterogeneous real-world axillary workflow. Subgroup analysis showed that the clinically node-negative upfront SLNB subgroup is the most appropriate population for comparison with published ICG-only SLNB literature, whereas pooled outcomes should be interpreted as implementation-level data rather than sentinel-node-specific performance metrics. ICG-guided sentinel mapping was attempted in all patients and was successful in 259/260 (99.6%) cases according to operative records. In the predefined complete-case node-focused cohort (n = 230), lymph node yield was consistent with the final axillary procedure performed, and nodal metastasis was identified in 25.2% of patients. Given the retrospective design, heterogeneous case mix, and lack of an internal comparator, these results should be interpreted as real-world feasibility data rather than formal diagnostic validation. Prospective multicentre studies with predefined accuracy endpoints and oncologic follow-up are needed to further clarify the role of ICG-only strategies in contemporary axillary staging.

## Figures and Tables

**Table 2:** Axillary procedure distribution, lymph node yield, nodal metastasis, and mapping success within the study cohort.

Outcome	Value
**Axillary procedure performed (n = 230)**	
• Sentinel node biopsy	166 (72.2%)
• Targeted axillary dissection	4 (1.7%)
• Axillary lymph node dissection	60 (26.1%)
**Number of lymph nodes removed (overall), median (IQR)**	4 (3–7)
**Node yield by axillary procedure, median (IQR)**	
• Sentinel node biopsy	4 (3–5)
• Targeted axillary dissection	6 (4.75–7)
• Axillary lymph node dissection	11 (7.75–18)
**Any nodal metastasis (≥1 metastatic node)**	58/230 (25.2%)
**Metastatic node count among positive cases, median (IQR)**	2 (1–3)
**Successful sentinel mapping (operative record, study population n = 260)**	259/260 (99.6%)

**Table 4:** Reoperation patterns, postoperative complications, revision surgery, and residual nodal burden following axillary surgery.

Outcome	Value
**Reoperation for ALND**	14/230 (6.1%)
**Reoperation after SLNB**	14/166 (8.4%)
**SLNB-positive patients**	30
**SLNB-positive proceeding to ALND**	14/30 (46.7%)
**ALND cases with nodal data available**	13
**Residual metastatic nodes at ALND**	6/13 (46.2%)
**Postoperative complications**	36/230 (15.7%)
• Seroma	22
• Bleeding	8
• Inflammatory Wound Complications	6
**Revision surgery**	33/230 (14.3%)
• **Reoperation for ALND/axillary management**	14
• **Non-ALND revision surgery**	19
• **Bleeding/Haematoma revision**	6
• **Inflammatory wound revision**	1
• **Seroma revision (with drain placement)**	12
**Final Resection margins**	All R0

**Table 5:** Exploratory multivariable logistic regression analysis of factors associated with nodal metastasis in the predefined complete-case–cohort.

Predictor	Adjusted OR (95% CI)	*p*-Value
Age (per year)	**0.99 (0.97–1.02)**	**0.523**
Imaging tumour size (per mm)	**0.99 (0.96–1.02)**	**0.675**
Grade (G3 vs. G2)	**2.83 (0.98–8.17)**	**0.055**
Luminal B vs. Luminal A	**1.14 (0.54–2.41)**	**0.740**
HER2-enriched vs. Luminal A	**0.41 (0.05–3.34)**	**0.406**
Triple-negative vs. Luminal A	**0.82 (0.20–3.33)**	**0.781**
Multifocal vs. unifocal	**1.10 (0.43–2.83)**	**0.842**
Suspicious LN (biopsied-positive)	**12.85 (3.98–41.52)**	**<0.001**
Suspicious LN (biopsied-negative)	**0.68 (0.18–2.54)**	**0.567**
Suspicious LN (not biopsied)	**15.58 (3.44–70.59)**	**<0.001**
Neoadjuvant therapy	**0.31 (0.11–0.87)**	**0.026**

## Data Availability

The data supporting the findings of this study are available from the corresponding author upon reasonable request. The data are not publicly available due to patient privacy and institutional ethical restrictions. The authors conducted literature research using PubMed and Google Scholar to identify relevant scientific publications referenced in this manuscript.

## Abbreviations

The following abbreviations are used in this manuscript:

ALND	Axillary Lymph Node Dissection
CI	Confidence Interval
ICG	Indocyanine Green
IQR	Interquartile Range
MRI	Magnetic Resonance Imaging
NAC	Neoadjuvant Chemotherapy
OR	Odds Ratio
SLNB	Sentinel Lymph Node Biopsy
SPY-PHI	Fluorescence Imaging System (Stryker)
